# ASO Author Reflections: Toward Better Outcomes in Poorly Differentiated Thyroid Cancer: Evaluating Adjuvant Radiotherapy, Treatment-related Morbidity, and Future Therapeutic Directions

**DOI:** 10.1245/s10434-025-17725-8

**Published:** 2025-07-09

**Authors:** Pascal K. C. Jonker, Jan H. Koetje, Jia Feng Lin, Bruce G. Robinson, Schelto Kruijff, Mark S. Sywak

**Affiliations:** 1 University of Sydney Endocrine Surgical Unit, Royal North Shore Hospital, Northern Sydney Local Health District, St Leonards, Australia; 2 Department of Surgery, University Medical Center Groningen, University of Groningen, Groningen, The Netherlands; 3 Department of Endocrinology, Royal North Shore Hospital, Northern Sydney Local Health District, St Leonards, Australia; 4 Northern Clinical School, Sydney Medical School, Faculty of Medicine and Health, University of Sydney, Sydney, NSW Australia

## Past

Poorly differentiated thyroid cancer (PDTC), comprising up to 8% of thyroid cancers, has a poor prognosis, with deaths predominantly caused by locoregional recurrence (18%) or distant metastasis (85%).^1,2^ The role of postoperative radiotherapy remains uncertain due to limited evidence regarding its impact on local and regional disease control. In addition, comprehensive reporting of treatment-related complications remains insufficient. Similarly, the benefit of ^131^I is debated, influenced by inconsistent survival outcomes, varied definitions of refractory disease, and diverse treatment protocols. Finally, factors associated with disease specific survival are unknown.

## Present

This study evaluated the impact of postoperative radiotherapy (IMRT) on local disease control in patients with resectable PDTC classified by Turin criteria.^2^ Additionally, a comprehensive overview of treatment-related morbidity, the prevalence of ^131^I-refractory disease and factors influencing disease-specific survival were assessed. Adjuvant IMRT improved 5-year local disease control (100% versus 17.5%), altough it led to increased incidence of mild complications. Patients receiving adjuvant IMRT underwent more frequent TKI treatment. ^131^I-refractory disease developed in 62.7% of patients within 13 months and was more frequent among non-survivors (86.6% versus 52.8%). Positive resection margins and extrathyroidal extension correlated with reduced survival in univariate analysis but lost significance when evaluated in multivariate regression. These findings indicate that postoperative IMRT could effectively reduce recurrence in the thyroid bed for patients with positive margins, however this also may be partially linked to the administration of Lenvatinib. Although surgery and systemic therapy exhibit relatively low morbidity, IMRT treatment carries a higher risk of mild complications. The high incidence of ^131^I-refractory disease emphasizes PDTC’s aggressive behavior, and factors affecting disease-specific survival remain uncertain. Although the development of radio-iodine refractory disease is a common problem in PDTC, the addition of adjuvant IMRT to the thyroid bed in selected patients improves local disease control. We believe that this multimodality approach reduces the likelihood of symptomatic local recurrence in the thyroid bed and avoids the challenging problem of asphyxiation which can occur in uncontrolled local recurrence.

## Future

Improving outcomes for patients with PDTC requires enhanced understanding of tumor biology, standardization of current treatments, and development of novel therapies. These challenges are similar to the ones faced in the treatment of anaplastic thyroid cancer (ATC).^4,5^ International consortia could address critical clinical questions through prospective databases and trials, evaluating therapies like IMRT, ^131^I, tyrosine kinase inhibitors and immune checkpoint inhibitors. Although genomic studies exist, comprehensive integrative analyses linking biology to clinical outcomes remain limited. Collaborative research could significantly accelerate clinical trials, optimize existing treatments, introduce new therapeutic options, and ultimately enhance patient outcomes in rare and aggressive forms of thyroid cancer.^5^
